# CNN3 Regulates Trophoblast Invasion and Is Upregulated by Hypoxia in BeWo Cells

**DOI:** 10.1371/journal.pone.0103216

**Published:** 2014-07-22

**Authors:** Sarah Appel, Janina Ankerne, Jan Appel, Andre Oberthuer, Peter Mallmann, Jörg Dötsch

**Affiliations:** 1 Department of Pediatrics and Adolescent Medicine, University of Cologne, Cologne, Germany; 2 Neonatal and Pediatric Intensive Care Unit, University of Cologne, Children's Hospital, Cologne, Germany; 3 Department of Obstetrics and Gynecology, Cologne University, Cologne, Germany; Chinese Academy of Sciences, China

## Abstract

CNN3 is an ubiquitously expressed F-actin binding protein, shown to regulate trophoblast fusion and hence seems to play a role in the placentation process. In this study we demonstrate that CNN3 levels are upregulated under low oxygen conditions in the trophoblast cell line BeWo. Since hypoxia is discussed to be a pro-migratory stimulus for placental cells, we examined if CNN3 is involved in trophoblast invasion. Indeed, when performing a matrigel invasion assay we were able to show that CNN3 promotes BeWo cell invasion. Moreover, CNN3 activates the MAPKs ERK1/2 and p38 in trophoblast cells and interestingly, both kinases are involved in BeWo invasion. However, when we repeated the experiments under hypoxic conditions, CNN3 did neither promote cell invasion nor MAPK activation. These results indicate that CNN3 promotes invasive processes by the stimulation of ERK1/2 and/or p38 under normoxic conditions in BeWo cells, but seems to have different functions at low oxygen levels. We further speculated that CNN3 expression might be altered in human placentas derived from pregnancies complicated by IUGR and preeclampsia, since these placental disorders have been described to go along with impaired trophoblast invasion. Our studies show that, at least in our set of placenta samples, CNN3 expression is neither deregulated in IUGR nor in preeclampsia. In summary, we identified CNN3 as a new pro-invasive protein in trophoblast cells that is induced under low oxygen conditions.

## Introduction

During human placentation, fetal trophoblast cells differentiate into an invasive and a non-invasive phenotype. The non-invasive cells which include the syncytiotrophoblast and the villous cytotrophoblast form chorionic villi. Some of them attach to the decidua (so called anchoring villi), and the cytotrophoblast at the site where the villous is attached to the decidua proliferates and builds a cell column. Here cells differentiate into the invasive extravillous trophoblast and start to invade the maternal tissue: the interstitial extravillous trophoblast reaches the decidua and the myometrium, whereas the endovascular extravillous trophoblast moves to the spiral arteries [1], [2]. To protect the mother, the invasion process has to be under a strict control and it is important that trophoblast cells are never proliferative and invasive at the same time. Both the interaction of the trophoblast cell with maternal immune cells [3], [4] and O_2_ levels in the developing placenta [5], [6] are important factors regulating the invasion process. It is well accepted that O_2_ levels are low in the developing placenta, displaying 17.9 mm Hg of partial oxygen pressure in the tissue up to week 8–10 of gestation. Around week 12–13, partial oxygen pressure rises to 39.6 mm Hg [7]. As for the O_2_ levels, controverse data exist as to whether hypoxic conditions inhibit or promote trophoblast invasion [8], [9], [10].

Several proteins are known to participate in the regulation of cell migration. One of them is CNN3, a member of the Calponin family. Calponin proteins exist in 3 different isoforms, deriving from 3 different genes: CNN1 (h1/basic calponin) [11], CNN2 (h2/neutral calponin) [12] and CNN3 (h3/acidic calponin) [13]. They consist of a conserved N-terminal Calponin homology (CH) domain, followed by three calponin-like repeats (CLIK) which serve as actin-binding sites and a variable C-terminus [14], [15], [16]. All Calponin proteins are involved in the regulation of cell migration, however, isoform specific differences exist [17], [18], [19], [20]. Recently, the group of Shibukawa et al. described that CNN3 participates in the regulation of trophoblast fusion by actin cytoskeleton rearrangement, and this is dependent on phosphorylation events of CNN3 [21]. This suggests an important role for this protein in the placentation process. Whether CNN3 is also involved in regulatory pathways besides trophoblast fusion in the placenta and how its expression is regulated in this tissue is not known so far. The aim of this study was to reveal if CNN3 is capable of modifying trophoblast invasion and if CNN3 levels are influenced by oxygen levels.

## Material and Methods

### Cell culture and transfection

The choriocarcinoma cell line BeWo (DSMZ, Germany) was maintained in DMEM/F-12 without Phenol red (Invitrogen, Germany) supplemented with 10% FBS (Invitrogen) and 1% Pen/Strep (Invitrogen). For siRNA experiments, cells were seeded at 5×10^5^/60 mm culture dish and transiently transfected using Lipofectamine2000 transfection reagent (Invitrogen) according to the manufacturer's protocol. A mix of 4 different siRNA sequences against human CNN3 (SMARTpool human CNN3) was purchased from Thermo Scientific (Germany). As control, a mix of 4 different non-targeting siRNA sequences was used (Thermo Scientific, non-targeting siRNA Pool #2). For hypoxia experiments, cells were incubated in a hypoxic chamber (O_2_ control glove box for tissue culture; COY Laboratory Products Inc., USA) and exposed to a 1% oxygen atmosphere, whereas normoxic cells were kept at regular atmosphere (20.9% oxygen). For HIF-1alpha stabilization experiments, BeWo cells were serum starved for 16 h and then treated with either PBS as control or with 200 µM CoCl_2_ (Sigma) for 6 h in serum free medium.

### RNA expression analysis

For Reverse transcriptase-PCR, total RNA was prepared using the TRIzol reagent (Invitrogen) according to the manufacturer's recommendations. 1 µg total RNA was reverse transcribed with the MMLV Reverse Transcriptase (Promega, Germany) using Oligo-dT oligonucleotides and random hexamer oligonucleotides (Promega). Expression of human CNN3 was determined by TaqMan Real-Time PCR analysis with oligonucleotides #4351372 purchased from Applied Biosystems (Germany). The following oligonucleotides and probes (MWG, Germany) were used for detecting MMP-2 and MMP-14 mRNA levels: MMP-2 for 5′-TGTGACGCCACGTGACAAG, rev 5′- CCAGTATTCATTCCCTGCAAAGA, probe 5′- CCACATTCTGGCCTGAGCTCCCG; MMP-14 for 5′- CCGGCCTTCTGTTCCTGAT; rev 5′- CCAGCGCTCCTTGAAGACA, probe 5′- CCTATGGGCCCAACATCTGTGACGG. Expression of genes was normalized to expression of Glycerinaldehyd-3-phosphat-Dehydrogenase (GAPDH) for 5′- CCCATGTTCGTCATGGGTGT, rev 5′- TGGTCATGAGTCCTTCCACGATA, probe 5′- CTGCACCACCAACTGCTTAGCACCC; Hypoxanthin-Phosphoribosyl-Transferase (HPRT) for 5′- CCGGCTCCGTTATGGC, rev 5′- GGTCATAACCTGGTTCATCATCA, probe 5′- CGCAGCCCTGGCGTCGTGATTA, beta-2-Microglobulin (b2MG) for 5′- TGACTTTGTCACAGCCCAAGATA, rev 5′- CCAAATGCGGCATCTTC, probe 5′- TGATGCTGCTTACATGTCTCGATCCCA and beta-actin for 5′- GATGGCCACGGCTGCTT, rev 5′- ACCCTCATTGCCAATGGT, probe 5′- CTACGAGCTGCCTGACGGCCAGG [22]. The probes were modified with FAM at the 5′ and TAMRA at the 3′ end. TaqMan PCR reagents (Platinum, Quantitative PCR SuperMix-UGD with ROX, Invitrogen) were applied according to the manufacturer's protocol. 2.5 µl of cDNA was used as template in a total reaction volume of 25 µl. The thermocycler parameters were 50°C for 2 min, 95°C for 10 min, followed by 40 cycles of 95°C and 60°C for 1 min, and the *7500 Real Time PCR System* from Applied Biosystems was used. The results of the Real-Time PCR analysis were calculated based on the ΔΔCt–method and expressed as fold change of mRNA expression compared to the corresponding control group (1.0-fold induction).

### SDS-PAGE and Western Blot analysis

Cells were lysed in isotonic lysis buffer containing 10 mM NaPO_4_ pH 8.0, 140 mM NaCl, 3 mM MgCl_2_, 1 mM dithiothreitol, 0.5% Nonidet-P40 and 1× protease inhibitor mix (Roche Diagnostics, Germany) and protein lysates were subjected to SDS-PAGE and transferred onto nitrocellulose membranes (Whatman, Germany) according to standard procedures. The membranes were incubated either with the rabbit polyclonal anti-CNN3 antibody (calponin 3 H-55; Santa Cruz Biotechnology, Germany) at 1∶2000 dilution, the mouse monoclonal anti-alpha-tubulin antibody (clone DM1A, Sigma-Aldrich, Germany) at 1∶2,000,000 dilution, the rabbit polyclonal anti-HIF-1alpha antibody (#NB100-449, Novus Biologicals, Germany) at 1∶1000 dilution, the rabbit polyclonal anti-SAPK/JNK antibody (#9252, Cell Signaling, Germany) at 1∶1000 dilution, the rabbit polyclonal anti-phospho-SAPK/JNK (Thr183/Tyr185) antibody (#9251, Cell Signaling) at 1∶1000 dilution, the rabbit polyclonal anti-p38 MAPK antibody (#9212, Cell Signaling) at 1∶1000 dilution, the rabbit monoclonal anti-phospho-p38 MAPK (Thr180/Tyr182) (D3F9) XP antibody (#4511, Cell Signaling) at 1∶1000 dilution, the rabbit polyclonal anti-p44/42 MAPK (Erk1/2) antibody (#9102, Cell Signaling) at 1∶2000 dilution, the rabbit monoclonal anti-phospho-p44/42 MAPK (Erk1/2) (Thr202/Tyr204) (D13.14.4E) XP antibody (#9102, Cell Signaling) at 1∶1000 dilution, the rabbit polyclonal anti-MMP-2 antibody (#4022, Cell Signaling) at 1∶500 dilution, the rabbit monoclonal anti-MMP14 antibody [EP1264Y] (#51074, abcam, Germany) at 1∶2000 dilution and the rabbit polyclonal anti-HPRT antibody (#10479, abcam) at 1∶1000 dilution over night at 4°C. As secondary antibodies, the horseradish-peroxidase (HPO)-linked secondary antibodies goat-anti-mouse-HPO and goat-anti-rabbit-HPO (Cell Signaling) were used at 1∶2000 dilution at room temperature for 1 h. The ECL Prime Western Blotting Detection Reagent (GE Healthcare, UK) was used to visualize protein bands with the Bio-Rad Molecular Imager ChemiDOC XRS+ Imaging System (Bio-Rad Laboratories) and densitometry was determined using the Image Lab software Version 4.0.1 (Bio-Rad Laboratories).

### Zymogel analysis

Cell culture supernatants were incubated with non-denaturating sample buffer (62.5 mM Tris-HCl pH 6.8, 10% glycerol, 2% SDS, 0.0025% Bromphenol blue) for 10 minutes at 37°C and loaded onto 10% SDS-polyacrylamide gels containing 0.1% gelatin. Following gel electrophoresis, SDS was removed by washing the gel once for 30 minutes in renaturation buffer (2.5% Triton X-100) and once more for 30 minutes in developing buffer (50 mM Tris-HCl pH 8, 0.2 M NaCl, 5 mM CaCl_2_, 0.02% Brij 35). The gel was developed over night at 37°C in developing buffer. For staining of the gel we used 0.1% Coomassie brilliant blue in 25% isopropanol and 10% acetic acid. White bands were analyzed with the Bio-Rad Molecular Imager ChemiDOC XRS+ Imaging System (Bio-Rad Laboratories) and densitometry was determined using the Image Lab software Version 4.0.1 (Bio-Rad Laboratories).

### Invasion assay

Invasion assay was performed with BeWo cells either transfected with control-siRNA or with siRNA against human CNN3. Alternatively, BeWo cells were treated either with DMSO as control, the MEK inhibitor U0126 (Cell Signaling) at 10 µM concentration or the p38 inhibitor SB203580 (Cell Signaling) at 21 nM concentration for the time of the invasion assay. For low oxygen experiments, cells were incubated in a hypoxic chamber and exposed to a 1% oxygen atmosphere, whereas normoxic cells were kept at regular atmosphere (20.9% oxygen). Transwell inserts with a pore size of 8 µm (BD Biosciences, Germany) were coated with 100 µg growth factor reduced matrigel (BD Biosciences) according to manufacturers' instructions. 48 h after transfection, cells were seeded at a density of 1×10^5^ in the upper chamber of the transwell insert in serum free medium. The lower chamber was filled with medium containing 10% serum as chemoattractant. Following incubation at 37°C and 5% CO_2_ for 24 h, cells on the upper side of the membrane were removed with cotton swabs, and then cells on the bottom side of the membrane were fixed with methanol and stained with hematoxilyn (Roth, Germany) and eosin-G solution (Roth). Invaded cells were counted under a microscope (for siRNA-CNN3 experiments: Axiovert 200, Zeiss, Germany; for all other experiments: Leica DM6000B, Leica, Germany) at 10x magnification in bright field.

### MTT proliferation assay

BeWo cells were plated at a density of 1×10^4^ cells/well in a 96 well plate and cultured for 24 h in the presence of either DMSO, 10 µM U0126 or 21 nM SB203580. For CNN3 knockdown experiments, cells were transfected with siRNA-control or siRNA-CNN3, cultured for 48 h and then plated onto a 96 well plate for 24 h. For hypoxia experiments, cells were incubated at a 1% oxygen atmosphere. The MTT proliferation assay was performed according to the manufacturers' protocol (ATCC 30-1010K, LGC Standards GmbH, Germany).

### Placenta samples

Placentas from either control or preeclamptic patients were collected in the Department of Obstetrics and Gynecology, University Hospital Cologne. All deliveries were planned caesarean sections. Preeclamptic parameters were as follows: systolic blood pressure >140 mm Hg and diastolic blood pressure >90 mm Hg as well as proteinuria with >300 mg in 24 h urine, both symptoms first occurring after gestational week 20 [23]. Criterions for exclusions were multiple pregnancies, diabetes mellitus, cardiac insufficiency, Chorioamnionitis, HELLP (hemolysis, elevated liver enzymes, low platelet count) and HIV infection. We collected 17 preeclampsia samples (gestational week 32.2+2.8 (SD)) and 9 control samples (gestational week 35.8+2.9 (SD)). The study was approved by the ethical committee of the University of Cologne and has been conducted in accordance with the principles of the Declaration of Helsinki (revised in 2000). Written consent was collected from all subjects prior to the study. Placenta samples from a tissue collection of a previous study [24], [25], [26] were used for the analysis of CNN3 expression in intrauterine growth restriction (IUGR) and average for gestational age (AGA) placentas, containing 39 IUGR samples (gestational week 35.3+2.6 (SD)) and 31 AGA samples (gestational week 36.6+2.8 (SD)) as control.

For sample preparation, placentas were dissected to remove the amniotic membranes and maternal decidua. The residual layer, containing the villous trees, was washed in PBS to remove the blood. Then, a sample from the middle region was excised, quick frozen on dry ice and stored at -80°C. For immunofluorescence analysis, samples from the middle region containing villous trees where embedded in Tissue-Tek O.C.T. Compound (Sakura Finetek Europe B.V., Netherlands), quick frozen and then stored at -80°C.

### Immunofluorescence analysis

10 µm tissue slides were blocked and permeabilized with 5% goat serum and 0.2% Triton X-100 and subsequently stained with the rabbit-anti-CNN3 antibody at 1∶50 dilution in Dako REAL Antibody Diluent (Dako, Germany) for 24 h at 4°C. As secondary antibody, the goat-anti-rabbit-Cy3 (Dako) was used at 1∶200 dilution for 2 h at room temperature in Dako REAL Antibody Diluent (Dako). To visualize actin filaments, the tissue slides were incubated with Alexa Fluor 488 Phalloidin (Molecular Probes, Germany) at 1∶5000 dilution for 15 min at room temperature. Nuclei were stained with DAPI (Molecular Probes) for 15 min at room temperature.

### Statistical analysis

All values in the text are reported as means + SEM. Differences between means were evaluated using a two-tailed Student's t-test. Significant differences were taken at the p<0.05 level.

## Results

### CNN3 regulates cell invasion

**Figure 1:**
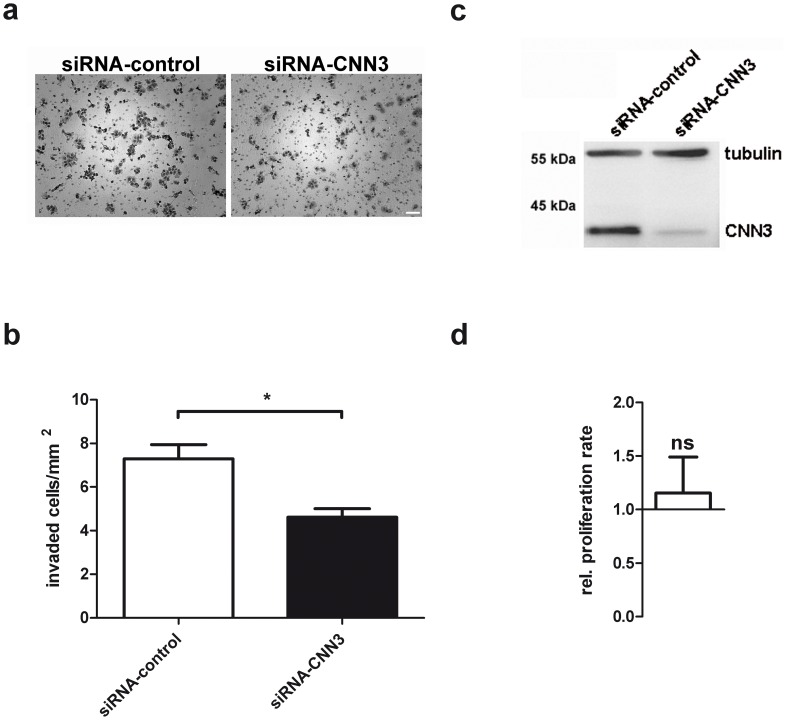
CNN3 regulates BeWo cell invasion. BeWo cells were transfected with siRNA against CNN3 or with control siRNA for 48(A) The cells were plated in serum free medium on transwell inserts coated with 100 µg matrigel at a density of 1×10^5^ cells/insert. In the lower chamber, medium containing 10% FCS was added as invasion stimulus. After 24 h, invaded cells were fixed and stained with H&E. (B) Five pictures from every membrane at 10x magnification were taken and cells were counted. Invaded cells/mm^2^ are displayed in the graph, whereas the white column represents control and the black column CNN3 knockdown cells. n = 3. (C) An aliquot of the cells for the invasion assay was kept for Western Blot studies to verify CNN3 knockdown. Total protein lysates were extracted from the cells and examined via Western Blot. CNN3 was detected on the membrane using a specific antibody. For normalization, tubulin-alpha was detected on the same membrane. (D) For the MTT Proliferation assay, cells were transfected with siRNA-CNN3 for 48 h and then cultured in a 96-well plate for 24 h. Then the proliferation rate was determined using the MTT proliferation kit (ATCC). Relative proliferation rates for CNN3-siRNA transfected cells (white column) are plotted compared to the control-siRNA transfected cells (set as 1). n = 3.

We first examined if cells lacking CNN3 display an altered invasion capability. Therefore, BeWo cells were transfected with siRNA against CNN3 or with non-coding control-siRNA. After 48 h, cells were seeded onto transwell inserts coated with matrigel. As chemoattractant, 10% serum was added to the culture medium in the outer chamber. To verify CNN3 downregulation, a sample of the cells 48 h after siRNA-transfection was analyzed via Western Blotting (Fig. 1c). 24 h after seeding cells onto the transwell insert, cells invaded through the matrigel and migrated to the bottom of the transwell insert were stained with H&E and counted under a microscope. Of the control cells, 7.3 cells/mm^2^ migrated through the matrigel and the pores towards the serum. On the other hand, CNN3 knockdown cells migrated less, counting only 4.6 cells/mm^2^ (Fig. 1a and b). This effect is not due to an impaired cell proliferation of siRNA-CNN3 transfected cells, as shown by a MTT proliferation assay (Fig. 1d). These data demonstrate that CNN3 plays a role in trophoblast cell invasion.

### CNN3 does not regulate MMP-2 and MMP-14 levels

**Figure 2:**
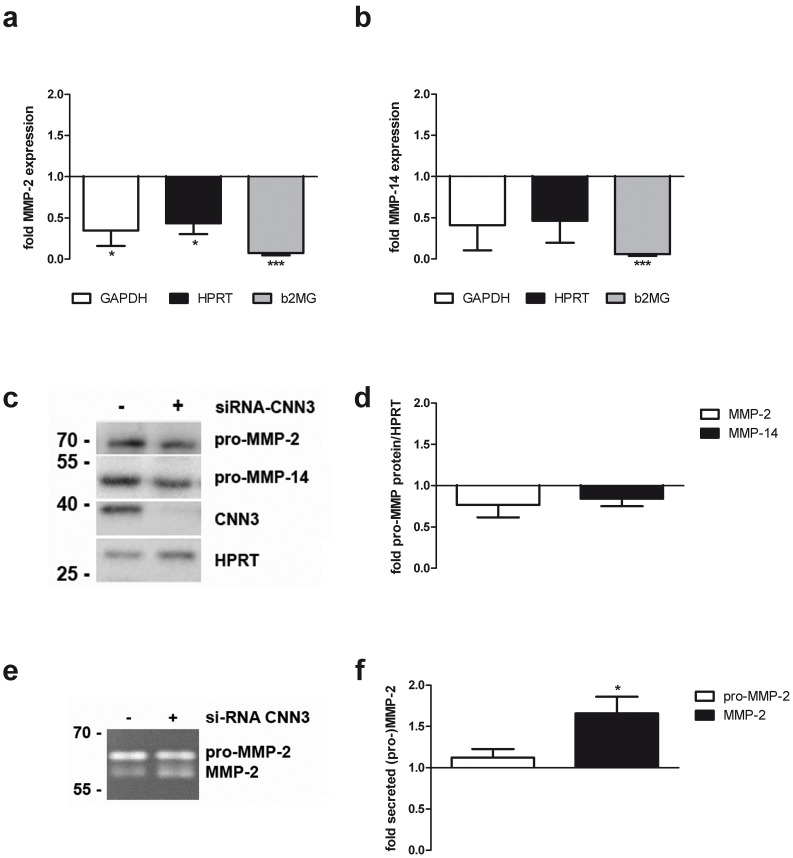
CNN3 does not regulate MMP-2 and MMP-14 levels in BeWo cells. CNN3 expression was knocked down in BeWo cells by transfection with specific siRNA. 48(A) and (B) A quantitative Real-Time-PCR assay was performed to determine MMP-2 (a) and MMP-14 (b) mRNA expression levels. Expression was normalized to the expression of the housekeeping genes GAPDH (white column), HPRT (grey column) and b2MG (black column). Expression levels of control cells were set as 1 and only levels for CNN3 knockdown cells are plotted in the graph. n = 3. (C) To measure MMP-2 and MMP-14 protein levels, protein lysates from either control or CNN3 knockdown cells were analyzed via Western Blotting. For normalization, HPRT was detected on the same membrane. (D) The intensity of the protein bands was densitometrically measured and MMP-2/HPRT and MMP-14/HPRT levels of CNN3 knockdown cells are plotted in the graph. The MMP-2/HPRT and MMP-14/HPRT levels in control cells were set as 1. n = 5. (E) We also detected pro-MMP-2 (upper band) and MMP-2 (lower band) levels in the supernatant of control or siRNA-CNN3 transfected cells by using a gelatin Zymogel. (F) Densitometric analysis of the bands was performed. Pro-MMP-2 (white column) and MMP-2 (grey column) levels in the control cells were set as 1 and only the levels in CNN3 knockdown cells are displayed in the graph. n = 10.

Since CNN3 is promoting trophoblast invasion trough a matrigel, we wanted to test the hypothesis that CNN3 is capable of altering the expression of proteases involved in those invasion processes. Hence, CNN3 expression was downregulated using siRNA against the CNN3 transcript and decreased CNN3 protein levels were confirmed in Western Blot studies (Fig. 2c) after 72 h. The expression level of MMP-2 and MMP-14 was analyzed in quantitative Real-Time PCR studies 72 h after siRNA transfection. In order to avoid serum effects on MMP expression, we incubated the transfected cells with serum free medium 24 h before harvesting the cells. As shown in Fig. 2a and b, CNN3 knockdown cells express significantly less MMP-2 and MMP-14 compared to control cells. But when we determined the protein level of MMP-2 and MMP-14 on a Western Blot, we saw the same tendency though the differences were not significant (Fig. 2c and d). We also performed a Zymogel analysis in order to measure the amount of secreted MMP-2 in BeWo cells expressing less CNN3 due to siRNA transfection. Here, the amount of the pro-MMP-2 form was not altered between control versus knockdown cells. To our surprise, the levels of active MMP-2 were even rather elevated in CNN3 deficient cells (Fig 2e and f).

In summary, CNN3 does not seem to regulate MMP-2 and MMP-14 protein levels, suggesting that other mechanisms involved in cell invasion are affected by CNN3.

### CNN3 activates ERK1/2 and p38, kinases that are involved in trophoblast invasion

**Figure 3:**
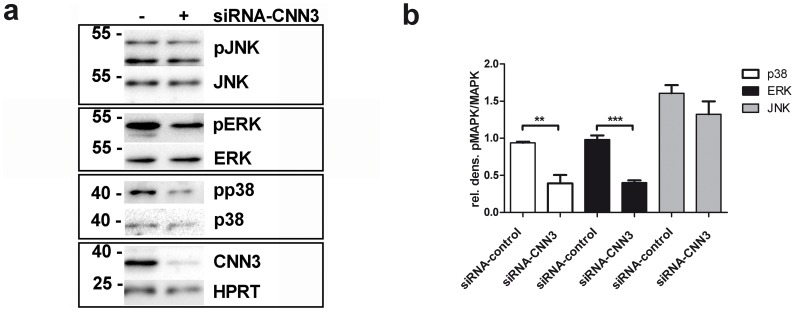
CNN3 activates ERK1/2 and p38. BeWo cells were transfected either with control or with CNN3-siRNA for 48 h, then serum starved for 24 h and finally harvested. Proteins were extracted from the cells and examined on a Western Blot. (A) Total ERK1/2, phospho-ERK1/2 (pERK); total p38, phospho-p38 (p38); total JNK and phospho-JNK (pJNK) levels were detected. To verify CNN3 knockdown, the protein was detected on the membrane using a specific antibody. For normalization, the membrane was stained for HPRT. (B) Densitometric analysis was performed and pERK/ERK, pp38/p38 and pJNK/JNK levels in either control or CNN3 knockdown cells are plotted in the graph. n = 3.

Since CNN3 does not regulate MMP-2 and MMP-14 levels, we suppose that other mechanisms being involved in BeWo invasion are affected by CNN3. Members of the MAPK family have been connected to migratory events in several cell types [27]. Since CNN3 regulates ERK1/2 phosphorylation in fibroblast cells [20], we speculated that CNN3 might also regulate trophoblast invasion by activating one of the MAPKs p38, JNK or ERK1/2. To test this assumption, CNN3 was knocked down in BeWo cells and total protein extracts were analysed for MAPK activity in a Western Blot assay. As can be seen in Fig. 3a and b, a lack of CNN3 results in a decreased activity of both p38 and ERK1/2, whereas JNK activity was not affected in this experimental setting.

**Figure 4:**
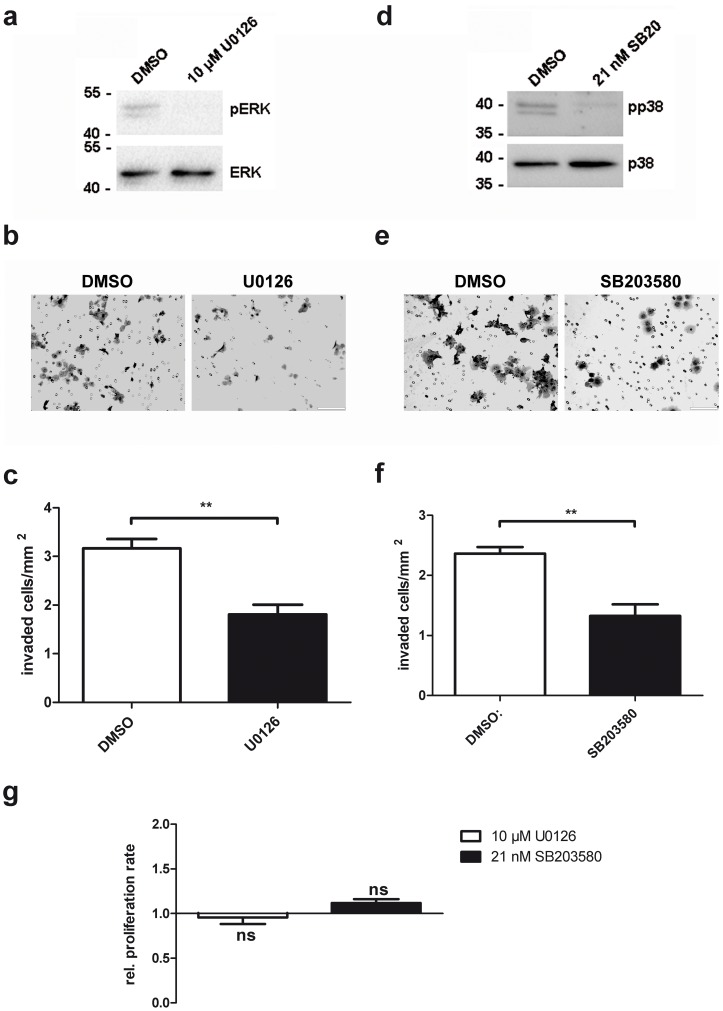
The MAPK ERK1/2 and p38 promote trophoblast invasion. (A) and (D) BeWo cells were serum starved for 16 h, pretreated with either DMSO as control, 10 µM of the MEK inhibitor U0126 or with 21 nM of the p38 inhibitor SB203580 in serum free medium. Then, cell culture medium containing 10% FCS was added to the cells, containing either DMSO as control, 10 µM U0126 or 21 nM SB203580. 24 h later, cells were harvested and proteins were analysed by Western Blotting. Phospho-ERK1/2 (pERK), total ERK1/2, phospho-p38 (pp38) and p38 signals were detected on the membranes. n = 3. (B) and (E) BeWo cells were plated onto transwell inserts coated with 100 µg matrigel at a density of 1×10^5^ cells/insert in serum free medium, containing either DMSO as control, 10 µM U0126 (B and C) or 21 nM SB203580 (E and F). The lower chamber was filled with medium supplemented with 10% FCS as chemoattractant. 24 h later, cells that invaded through the matrigel and migrated to the membrane side facing the lower chamber were fixed and stained with H&E. n = 3. (C) and (F) From each membrane, five pictures were taken at 10x magnification and cells were counted. The number of invaded cells/mm^2^ is plotted in the graphs. n = 3 for U0126 and n = 4 for SB203580. (G) For the MTT Proliferation assay, cells were cultured in the presence of either DMSO as control, 10 µM U0126 or 21 nM SB203580 for 24 h. Then the proliferation rate was determined using the MTT proliferation kit (ATCC). Relative proliferation rates for U0126 (white column) and SB203580 (black column) are plotted compared to the DMSO control (set as 1). n = 3.

To test if ERK1/2 and p38 are involved in trophoblast cell invasion, we performed matrigel invasion studies with BeWo cells either treated with DMSO as control or with the appropriate inhibitors. The MEK inhibitor U0126 was used to block ERK1/2 activation, whereas SB203580 was used to inhibit p38 activity. The efficacy of these inhibitors was corroborated in a Western Blot (Fig. 4a and d). Indeed we saw that both blocking ERK1/2 and p38 phosphorylation in BeWo cells also blocks their invasive potential (Fig. 4b,c and e,f). This was not due to inhibited cell proliferation by the MAPK inhibitors within 24 h, as can be seen in the MTT proliferation assay (Fig. 4g).

In summary, CNN3 might promote trophoblast invasion by activating the MAPKs ERK1/2 and p38.

### Hypoxic conditions upregulate CNN3 protein levels

**Figure 5:**
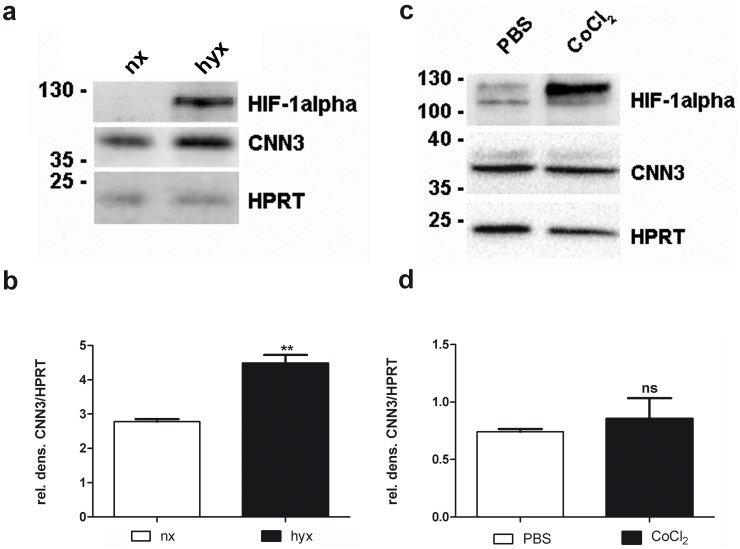
Hypoxic conditions upregulate CNN3 in BeWo cells. BeWo cells were cultured either under normoxic or under hypoxic conditions for 24(A) Total protein lysates were examined with the Western Blot technique to detect CNN3 protein levels. For normalization, HPRT was stained on the same membrane. As hypoxia marker, HIF-1 alpha was detected as well. (B) Protein bands of CNN3 and HPRT were densitometrically measured on the Western Blot membrane and CNN3/HPRT levels are plotted in the graph (white column: normoxia; black column: hypoxia). n = 3. (C) BeWo cells were serum starved for 16 h and then treated with either PBS as control or 200 µM CoCl_2_ for 6 h in serum free medium. Then total protein was isolated and a Western Blot was performed. The HIF-1 alpha, CNN3 and HPRT protein was detected on the membrane with specific antibodies. (D) A densitometric analysis was performed to determine CNN3/HPRT levels (white column: normoxia; black column: hypoxia). n = 3.

To determine if CNN3 levels are altered upon a hypoxic stimulus, the choriocarcinoma cell line BeWo was incubated either under normoxic or under hypoxic conditions for 24 h. Cells were harvested, and total protein was isolated. Performing a Western Blot analysis, we saw that CNN3 protein levels are elevated after 24 h hypoxia (Fig. 5a and b). As a control for hypoxia, we also detected HIF-1alpha on the Western Blot membrane, demonstrating that after 24 h the hypoxia-sensitive transcription factor was stabilized, resulting in higher protein levels and hence indicating hypoxic stress in the cell line (Fig. 5a). To examine if the transcription factor HIF-1alpha is responsible for the upregulation of CNN3 levels under hypoxia, we performed cell culture experiments with CoCl_2_ in order to stabilize the HIF-1alpha protein [28], [29]. However, CNN3 levels were unaltered upon CoCl_2_ treatment, suggesting that the hypoxia sensitive transcription factor is not involved in the upregulation of CNN3 levels under low oxygen conditions (Fig. 5c and d).

### Hypoxia stimulates cell invasion and p38 activity

**Figure 6:**
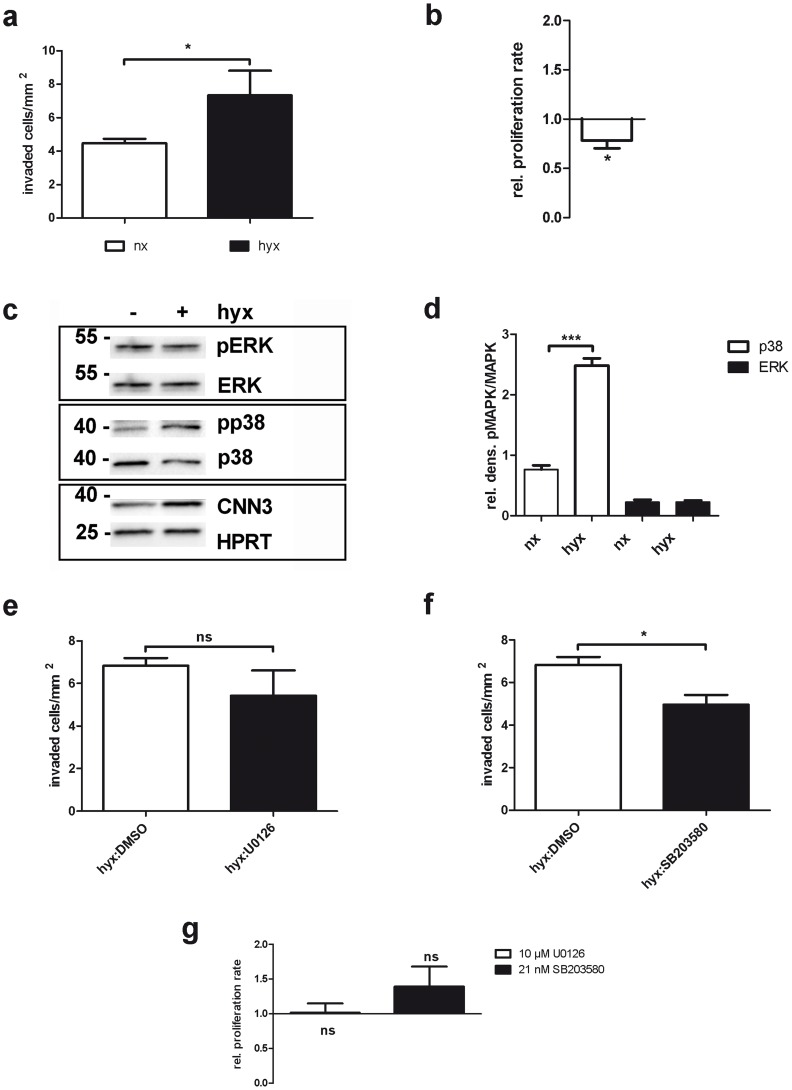
Hypoxia stimulates BeWo cell invasion and p38 activity. (A) BeWo cells were plated in serum free medium on transwell inserts coated with matrigel. In the lower chamber, medium containing 10% FCS was added as invasion stimulus. Then, cells were incubated for 24 h either at normoxic or at hypoxic conditions and at the end of the experiment, invaded cells were fixed and stained with H&E. Five pictures from every membrane at 10x magnification were taken and cells were counted. Invaded cells/mm^2^ are displayed in the graph, whereas the white column represents cells under normoxic and the black column represents cells under hypoxic conditions. n = 3. (B) For the MTT Proliferation assay, cells were cultured either at regular or at low oxygen levels for 24 h. Then the proliferation rate was determined using the MTT proliferation kit (ATCC). Relative proliferation rates for hypoxic conditions (white column) are plotted compared to the normoxic control (set as 1). n = 3. (C) BeWo cells were plated and serum starved for 16 h. Then they were either incubated at regular oxygen (- hyx) or at low oxygen (+ hyx) levels for further 24 h. Cell lysates were analysed for phospho-ERK1/2 (pERK), total ERK1/2 (ERK), phospho-p38 (pp38), total p38 (p38), CNN3 and HPRT protein levels on a Western Blot. (D) A densitometric analysis was performed to determine pp38/p38 (white columns) and pERK/ERK (black columns) ratios and plotted in a graph. n = 3. (E) An invasion assay under hypoxic conditions was carried out with either DMSO treated cells as control or with 10 µM U0126 treated cells. The number of invaded cells/mm^2^ is plotted in the graph. n = 3. (F) Shown is the analysis of n = 3 invasion assays under hypoxic conditions with either control cells (DMSO, white columns) or cells treated with 21 nM SB203580 (black columns). (G) BeWo cells were cultured in the presence of either DMSO as control, 10 µM U0126 or 21 nM SB203580 for 24 h. Then the proliferation rate was determined using a MTT proliferation kit (ATCC). Relative proliferation rates for U0126 (white column) and SB203580 (black column) are plotted in the graph compared to the DMSO control (set as 1). n = 3.

We next raised the question if the invasive potential of BeWo cells is altered under low oxygen conditions. Hence, invasion assays were perfomed under either a normoxic or a hypoxic atmosphere. As can be seen in Fig. 6a, significantly more cells migrated through the matrigel when cultured under hypoxia. The higher cell number was not due to higher cell proliferation rates under these conditions as can be seen in the MTT proliferation assay. Rather, cells proliferated significantly less under the low oxygen atmosphere (Fig. 6b).

To determine if ERK1/2 or p38 might play a role in the low oxygen induced trophoblast invasion, we performed Western Blots with cells either incubated under normoxia or hypoxia and probed for phospho-ERK1/2 and phospho-p38. While the activity of ERK1/2 did not change, p38 phosphorylation was significantly increased when incubated at low oxygen conditions (Fig. 6c and d).

Interestingly, the invasion potential of cells treated with a p38 inhibitor was significantly decreased under hypoxic conditions (Fig. 6f). The ERK1/2 inhibitor, on the other hand, did not have any significant effects on the number of invaded cells under hypoxia (Fig. 6e). Again, the lower number of invaded cells treated with the p38 inhibitor was not due to decreased cell proliferation rates (Fig. 6g).

In summary, the results indicate that p38 activity is increased and essential for cell invasion at low oxygen levels, while ERK1/2 seems not to be involved.

### CNN3 does not regulate trophoblast invasion under hypoxic conditions

**Figure 7:**
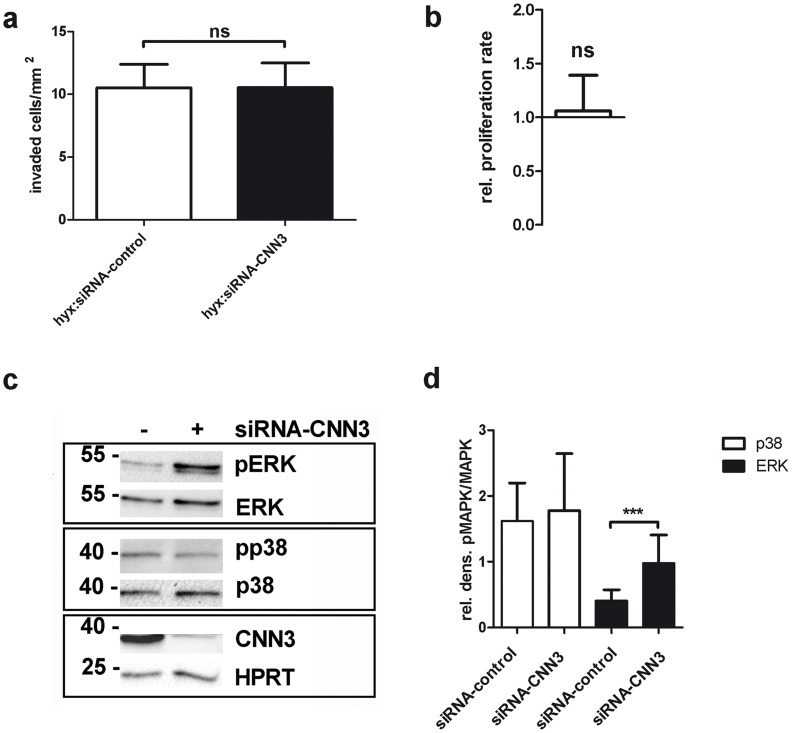
CNN3 does not promote cell invasion or MAPK activation under hypoxic conditions. (A) An invasion assay was performed with either siRNA-control transfected cells or with siRNA-CNN3 transfected cells at low oxygen levels. After fixation and staining of the invaded cells, pictures of the membrane were taken and the cell number/mm^2^ was determined and plotted in the graph. n = 3. (B) In a MTT proliferation assay, the proliferation rate of siRNA-CNN3 transfected BeWo cells compared to siRNA-control transfected cells under low oxygen levels was measured. Relative proliferation rates for CNN3 knockdown cells are plotted in the graph compared to the control (set as 1). n = 3. (C) siRNA-control or siRNA-CNN3 transfected BeWo cells were serum starved for 16 h and then incubated under hypoxic conditions. Whole cell lysates were analyzed on a Western Blot for phospho-ERK1/2 (pERK), total ERK1/2 (ERK), phospho-p38 (pp38), total p38 (p38), CNN3 and HPRT protein levels. (D) A densitometric analysis of n = 3 Western Blot membranes was performed and relative ratios of pp38/p38 (white columns) and pERK/ERK (black columns) levels are plotted in the graph.

The fact that CNN3 promotes trophoblast invasion in an assay under normoxic conditions (see Fig. 1) and that it is upregulated under hypoxic conditions (see Fig. 5) let us to the hypothesis that this protein might play a role in the regulation of trophoblast invasion under low oxygen conditions which can be found in the early placentation process. Hence, we performed a cell invasion assay with CNN3-knockdown cells under hypoxia. To our surprise, the downregulation of CNN3 had no effect on the number of invaded cells when exposed to low oxygen levels (Fig. 7a), indicating that CNN3 exerts its pro-invasive features only under normoxic conditions.

Assuming that CNN3 regulates cell invasion by activating ERK1/2 and/or p38, we speculated that CNN3 does not participate in the regulation of the invasion process under hypoxia due to different effects on the MAPK activities under these conditions. To test this hypothesis, we analyzed the impact of a CNN3 knockdown in BeWo cells cultured at a low oxygen atmosphere. Indeed, p38 activity was no longer affected by CNN3 depletion under these conditions. Moreover, ERK1/2 phosphorylation was increased in siRNA-CNN3 transfected cells under hypoxia (Fig. 7c and d) in contrast to reduced phospho-ERK1/2 levels upon CNN3 knockdown under normoxia (Fig. 3a and b).

Together these results indicate that CNN3 indeed modulates trophoblast invasive properties by activating ERK1/2 and/or p38, however only under normoxic conditions. Under hypoxia, CNN3 effects on MAPK activity are different and cell invasion is no longer regulated by the protein.

### The mRNA expression level of CNN3 is not deregulated in human samples from placentas of preeclamptic mothers and of mothers whose fetus suffered from IUGR

**Figure 8:**
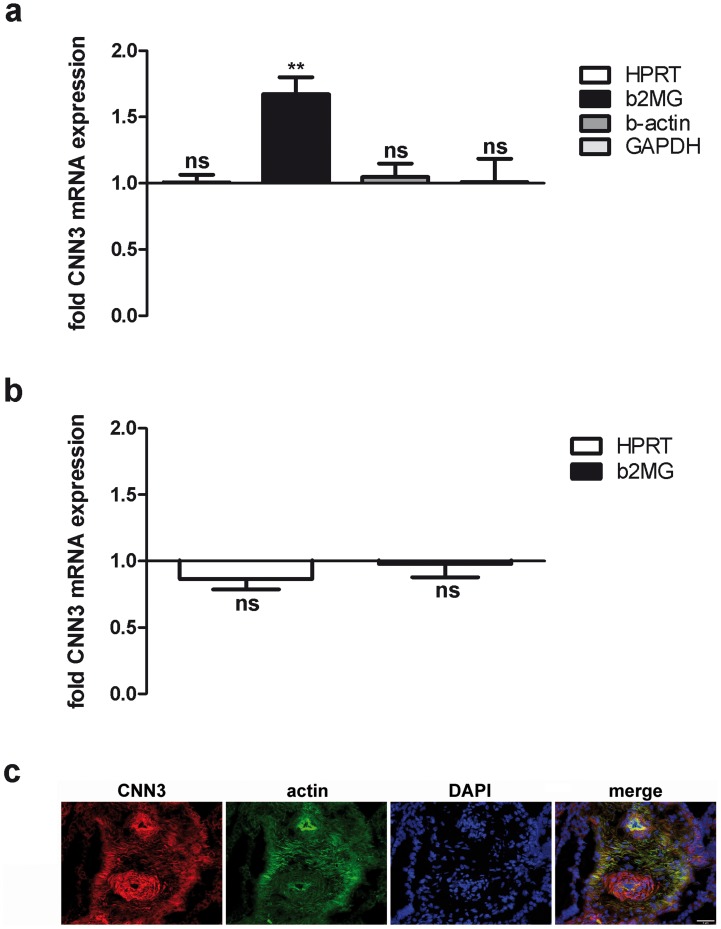
CNN3 mRNA expression is not altered in human placenta samples of preeclamptic mothers and of mothers whose fetus suffered from IUGR. Placentas were dissected to remove the amniotic membranes and the maternal decidua. The residual layer, containing the villous trees, was washed in PBS to remove the blood and a sample from the middle region was excised. Total RNA was purified and reverse transcribed into cDNA. Then, CNN3 mRNA expression was quantified in a Real-Time-qPCR analysis. For normalization, the expression level of several housekeeping genes was determined. (A) The placenta collection used for the study contained 17 preeclampsia samples (gestational week 32.2+2.8 (SD)) and 9 control samples (gestational week 35.8+2.9 (SD)) as control. For normalization, the genes HPRT, b2MG, b-actin and GAPDH were measured. (B) Placenta samples from a tissue collection of a previous study [24], [25], [26] were used for the analysis of CNN3 expression in 39 IUGR samples (gestational week 35.3+2.6 (SD)) and 31 AGA samples (gestational week 36.6+2.8 (SD)). For normalization, the mRNA levels of the housekeeping genes HPRT and b2MG were detected. (C) A cross section of a human placenta sample (from a healthy patient) containing villous trees was stained for CNN3 (anti-CNN3 antibody, red fluorescence), actin (phalloidin, green fluorescence) and nuclei (DAPI, blue fluorescence). Scale bar, 5 µm.

Placenta dysfunctions like IUGR or preeclampsia go along with an impaired trophoblast invasion [30], [31], [32], [33]. Hence, we speculated that CNN3 expression might be altered in placenta samples derived from patients giving birth to IUGR babies or suffering from preeclampsia. A total of 17 preeclamptic and 9 control placenta samples was examined for CNN3 mRNA expression via quantitative Real-Time PCR analysis. However, no significant differences were determined for CNN3 levels normalized to HPRT, b-actin and GAPDH (Fig. 8a). For CNN3 levels normalized to b2MG we saw a significant upregulation in preeclamptic compared to control samples (Fig. 8a), however we believe that this is an outlier and b2MG levels itself might be deregulated in preeclamptic placentas. For the analysis of CNN3 expression in IUGR, we used placenta samples from a tissue collection of a previous study [24], [25], [26], containing 39 IUGR samples and 31 AGA samples as control. As can be seen in Fig. 8b, there is no alteration in the CNN3 expression level in the IUGR samples compared to the AGA controls.

To ensure that CNN3 is expressed in trophoblast cells of the human placenta, we performed an immunofluorescence staining of a cross section of a human placenta sample from a healthy patient containing villous trees. Fig. 8c clearly shows that a CNN3 signal is present in villous cytotrophoblasts and also in endothelial cells of fetal blood vessels. However, no signal was detected in the syncytiotrophoblast layer. This is in accordance with data published by Shibukawa et al. 2010 [21]. Co-staining with phalloidin furthermore demonstrates that CNN3 is located at actin filaments in cytotrophoblast cells of human placenta.

## Discussion

It has long been known that the MAPKs JNK, ERK1/2 and p38 are involved in the regulation of cell migration. E.g., p38 promotes cell motility of smooth muscle cells [34] and fibroblast cells [35]. Substrates that have been identified to play a role in p38 dependent cell migration include proteins regulating actin dynamics like MAPKAP2/3 (MAPK-activated protein kinase 2/3, [36] and caldesmon [37]. Furthermore, ERK1/2 has been connected to motile events in several cell types such as fibroblasts [38] and smooth muscle cells [39]. The kinase modifies proteins like the Myosin light chain kinase (MLCK, [40]) and focal adhesion kinase (FAK, [41]), thereby influencing adhesion and actin dynamics as well. CNN3 activates p38 and ERK1/2 in trophoblasts and in parallel increases their invasive potential under normoxic conditions. Hence, it can be speculated that the cytoskeleton-associated protein regulates trophoblast invasion by modifying MAPK pathways leading to altered actin and adhesion dynamics. This is further corroborated by our finding that CNN3, under hypoxic conditions, neither activates p38 nor ERK1/2 and also fails to regulate cell invasion. On the other hand, CNN3 itself has been shown to modify actin stress fiber remodelling and hence, cell migration and contractile events in dermal fibroblasts [19]. Indeed, CNN3 is located at actin filaments in BeWo cells and regulates actin rearrangement [21]. We can therefore not exclude the possibility that the regulatory role of CNN3 in trophoblast invasion might be independent of MAPK signaling or involves other additional pathways where MAPKs do not play a role. This, however, has to be clarified in further experiments.

Matrix metalloproteinases (MMPs) play an important role in the invasion process of cells, since they degrade ECM proteins so that the cell is free to move. The activity of these proteases is controlled either by regulation of transcription [42] or by co-secretion and interaction with tissue inhibitors of MMPs (TIMPs) that regulate the activating proteolytic cleavage of the inactivating pro-domain of the MMPs in the extracellular space [43]. Invasive extravillous trophoblast cells do express several MMPs, including MMP-1, -2, -3, -7, -9, -11, -14 and -15 (overview in [2]). The fact that the CNN3 protein promotes cell migration through a matrigel let us speculate that proteases, important for this process, are regulated by CNN3. This assumption was based, amongst others, on the fact that transgelin (SM22) regulates MMP expression, and that this regulation is dependent on its type 3 Calponin homology domain [44]. However, when we tested for MMP-2 and MMP-14 levels in CNN3 knockdown cells, we saw a significant downregulation of the mRNA level, but no decrease on the protein level. In fact, the active form of MMP-2 was rather upregulated in the supernatant of CNN3 knockdown cells. This is inconsistent with our finding that a downregulation of CNN3 levels suppresses cell invasion and to this time point we can only speculate that MMP-2 seems not to be involved in BeWo cell invasion in our experimental setting. In summary, our data suggest that CNN3 does not exert its pro-invasive features under normoxic conditions by modulating MMP-2 or MMP-14 activity. We can, of course, not exclude that other proteases than MMP-2 or MMP-14, e.g. other MMPs or components of the urokinase pathway of plasminogen activation or protease inhibitors are affected by CNN3.

Hypoxia plays an important role in the regulation of trophoblast differentiation, proliferation and invasion, although data are conflicting. It is speculated that, depending on the trophoblast type and the time point of pregnancy, oxygen levels have various impacts on placental cells [10]. The fact that hypoxia is able to increase CNN3 levels in trophoblast cells indicates that CNN3 is involved in one of the pathways regulated by low oxygen levels. Several studies demonstrated that hypoxic conditions stimulate the migration of trophoblast cells. Most of the experiments have been performed *in vitro*, using either trophoblast cell lines, freshly isolated trophoblast cells or villous explants from human placentas [6], [45], [46], [47], [48]. Also, in this study we saw that BeWo cells display an increased invasive potential under low oxygen levels compared to normoxia. A study using a rat model corroborates these data *in vivo*, showing that maternal hypoxia results in an increased invasion of endovascular trophoblast cells [49]. Indeed, CNN3 knockdown impairs trophoblast invasion under normoxic conditions and we assumed that the protein especially participates in migratory processes in the early placenta, where trophoblast cells start to invade maternal tissues under hypoxic conditions. However, when we performed the invasion assay with CNN3 knockdown cells in the presence of low oxygen levels, no effect on cell invasion was visible. Hence, CNN3 seems to be involved in trophoblast migration, but not under hypoxic conditions. We therefore speculate that CNN3 is involved in other pathways than invasion regulated by low oxygen levels due to its upregulation under hypoxia. Previously published work describes a stimulation of trophoblast proliferation by hypoxic conditions [10]; however, any effect of CNN3 on the proliferative potential both under normoxic and hypoxic conditions was excluded in this study. More research is clearly needed to reveal CNN3's role in the presence of low oxygen levels.

A connection between an impaired trophoblast invasion and pregnancies complicated by IUGR and preeclampsia has been described [30], [31], [32], [33]. We therefore asked the question if the expression level of the pro-invasive factor CNN3 is altered in placentas derived from pregnancies complicated by IUGR or preeclampsia. However, in quantitative Real-Time PCR studies we were not able to confirm this hypothesis for IUGR placenta samples. We also detected no differences in CNN3 mRNA expression levels in preeclamptic compared to control placentas. This suggests that alterations in the CNN3 level are neither connected to the pathology of IUGR nor to preeclampsia. However, all placenta samples were taken from last trimester placentas, which might represent an inappropriate gestational time point for detecting changes in factors that are involved in early invasion processes. Right now we can only speculate that CNN3 has a physiological role in the early state rather than in the later state of pregnancy. A screening of CNN3 levels in early compared to term placentas could shed more light on this aspect. However, getting hand on human first trimester placenta samples is challenging and those samples are not available to our lab at the moment. Animal models would also be helpful to answer this question and we are currently planning mouse studies in order to learn more about CNN3's function in the placenta at different pregnancy time points. Finally, a clear statement whether a deregulated CNN3 activity is involved in the development of a pathological placenta or if the protein only plays an important physiological role during the embryonic phase cannot be made at this time point.

## Conclusion

In summary, we identified CNN3 as a new regulatory protein in trophoblast invasion under normoxic conditions. Moreover, CNN3 protein levels are increased under low oxygen conditions, suggesting a role for CNN3 in processes taking place in the early placentation process.
